# Is It Weird to Enjoy Solitude? Relationship of Solitude Capacity with Personality Traits and Physical and Mental Health in Junior College Students

**DOI:** 10.3390/ijerph17145060

**Published:** 2020-07-14

**Authors:** Pin-Hsuan Lin, Po-Yu Wang, Ying-Lien Lin, Shang-Yu Yang

**Affiliations:** 1 Department of Health and Beauty, Shu Zen Junior College of Medicine and Management, Kaohsiung 821, Taiwan; pinhsuan12@ms.szmc.edu.tw; 2 Department of Pediatric Emergency, Changhua Christian Children Hospital, Changhua 500, Taiwan; dama0115@yahoo.com.tw; 3 Department of Industrial and Information Management, National Cheng Kung University, Tainan 701, Taiwan; t10025023@gm2.nutn.edu.tw; 4 Department of Healthcare Administration, College of Medical and Health Science, Asia University, Taichung 413, Taiwan

**Keywords:** solitude capacity, personality traits, physical and mental health, college students

## Abstract

*Background*: Teenagers described as enjoying their own company have been claimed to have a weird personality and experience loneliness and negative emotions and have often been labeled with negative attributes. However, previous studies have provided a limited understanding of teenagers’ capacity for solitude. *Objectives*: The purpose of this study was to explore the correlations between teenagers’ capacity for solitude and both personality traits and physical and mental health. *Methods*: This study employed a cross-sectional research design and collected data from a junior college located in Taiwan using a structured questionnaire, which consisted of demographic questions, a solitude capacity scale, a personality trait scale, and a physical and mental health scale. *Results*: A total of 562 participants were recruited (age = 17.56 ± 1.58 years). The total score of the solitude capacity scale was significantly correlated with four elements of the personality traits subscale: neuroticism, extraversion, openness, and conscientiousness. The solitude capacity subscale (i.e., the solitude-coping subscale) showed significant correlations with two of the physical and mental health elements, i.e., anxiety and insomnia and severe depression. *Conclusions*: The results verified the correlations between capacity for solitude and personality traits and did not show a positive association with negative personality traits (i.e., neuroticism). Moreover, the solitude coping capacity correlated positively with anxiety levels and negatively with depression.

## 1. Introduction

Studies have associated solitude with a high level of suffering and loneliness in teenagers and maintained that people who enjoy their own company tend to experience negative emotions and difficulties in being accepted by the majority, and they are thus often labeled with negative attributes [1,2,3]. Most research has suggested that being alone inevitably results in loneliness (a sense of isolation), which can exert negative effects on people’s physical and mental health, particularly in their adolescence and even in early adulthood [4,5]. These particularly strong effects occur during adolescence possibly because teenagers wish for social acceptance and feel a particularly strong desire to belong [6]. However, Terrell-Deutsch [7] proposed an opposing viewpoint that loneliness and solitude are two separate concepts and that loneliness, rather than solitude, damages the health of teenagers. As such, teenagers who feel lonely do not necessarily lack social relationships [8]. In addition, some scholars have suggested that teenagers who are isolated do not necessarily feel lonely and argued that they might start to enjoy and capitalize on being alone once they realize the benefits and gain the ability to remain alone [9]. Nevertheless, studies have focused predominantly on the negative effects of spending time alone and of loneliness on teenagers and showed limited understanding of teenagers’ capacity for solitude [10,11,12,13].

Teenagers’ attitudes toward solitude are considered to reflect their adaptation to a particular developmental stage and to be related to their personality traits; however, the descriptions of these personality traits are often negative, like introverted melancholy [13,14]. Thus, the public has a negative impression of the " Solitary" individual. Most studies on teenagers’ personality traits have referred to the Big Five model [15] and divided personality into five dimensions: neuroticism (tendency to show negative emotions), extraversion (tendency to show positive emotions and be active in social interactions), openness (tendency to be curious and creative), agreeableness (tendency to promote and maintain an egalitarian relationship with others), and conscientiousness (tendency to act systematically, responsibly, and in an organized manner) [15,16]. Other studies have revealed that openness, conscientiousness, or traits indicating high emotional stability levels are associated positively with people’s preference for solitude, whereas agreeableness is associated negatively with such preferences [15,17]. Moreover, people with high levels of extraversion, agreeableness, and traits indicating a high level of emotional stability are less prone to loneliness when isolated; people with high levels of openness and conscientiousness easily experience negative emotions when isolated [18,19]. Long, et al. [20] conducted a survey on 320 college students and reported that people with a high level of introversion tended to experience negative emotions when they were alone (e.g., feeling lonely). Although studies have investigated the association between the attitude (preference) toward solitude and personality traits [15], the association between the capacity for solitude and personality traits remains unclear. The capacity for solitude can be considered an indicator of positive adaptation; therefore, understanding the relationship between teenagers’ capacity for solitude and their personality traits could be conducive to the improvement of such capacity in those with a low preference for solitude according to their personality traits [21]. It may also reduce the negative impression that solitude gives to the public.

Loneliness is a mental state experienced commonly during adolescence and occurs frequently when people are alone. People experience loneliness repeatedly when they are 18 years old, and such experiences decline with age [22,23]. Loneliness is directly associated with unfavorable physical and mental health during adolescence and early adulthood. For example, lonely teenagers are prone to physical conditions (e.g., headaches; [24]), sleep disorders (e.g., insomnia; [25,26]), social dysfunction [27], depression and low self-esteem [28], psychological distress [29], and suicide or self-harm ideation and attempts [23,30]. These conditions reflect the negative effects of loneliness on teenagers’ physical and mental health. Therefore, improving teenagers’ capacity for solitude might reduce the negative impact of loneliness, which accompanies the experience of being alone, on their mental and physical health [31]. However, because scholarly understanding of the effects of teenage students’ capacity for solitude on their physical and mental health is inadequate, research into the correlation between solitude capacity and physical and mental health is required. This study explored the correlations among capacity for solitude, personality traits, and physical and mental health in teenage students. Such correlations may provide insight to education institutions and parents for the mitigation of potential health risks in teenagers.

## 2. Materials and Methods

### 2.1. Study Participants

**Figure 1:**
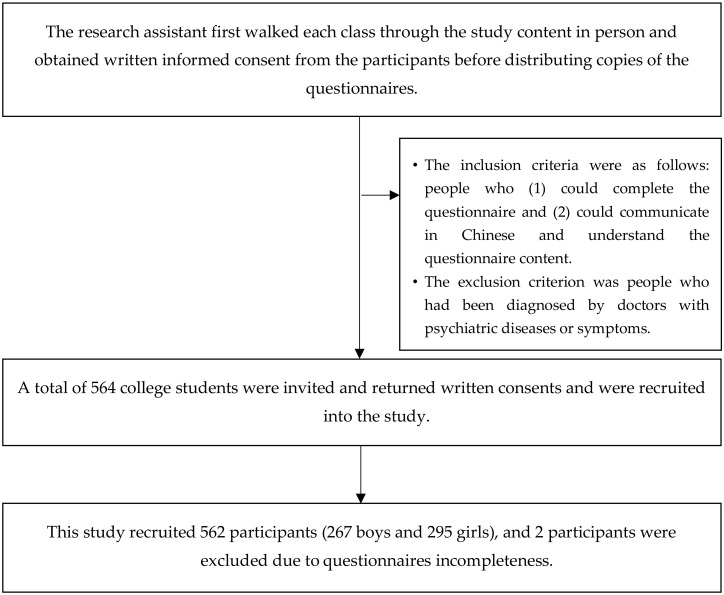
Flow chart of the selection of participants.

This study adopted a cross-sectional research design and administered a structured questionnaire to students in a junior college located in southern Taiwan for data collection. The research assistant first walked each class through the study content in person and obtained written informed consent from the participants before distributing copies of the questionnaires. For students younger than 18 years, consent from their guardian was required. The questionnaire survey lasted for three months, between May 1 and July 31 in 2019. The inclusion criteria were as follows: people who (1) could complete the questionnaire and (2) could communicate in Chinese and understand the questionnaire content. The exclusion criterion was people who had been diagnosed by doctors with psychiatric diseases or symptoms. A total of 564 college students were invited, returned written consents, and were recruited into the study. Ethical approval for the study was obtained from the National Cheng Kung University Human Research Ethics Committee (NCKU HREC-E-108-032-2). The data collection procedure is summarized in Figure 1.

### 2.2. Questionnaire

The questionnaire comprised six sections. Section 1 used demographical data including sex, age, body mass index, religious beliefs (yes or no), number of days of exercise per week (for exercise of at least 30 min duration), monthly allowance, relationship status, and place of residence (in a family home, college dormitory, or external rental property). Section 2 was composed of a solitude capacity scale for measuring the students’ ability to be in their own company. This scale was translated by Wu and Chen [32] from the Capacity to Be Alone Scale devised by Larson and Lee [33] and was composed of two subscales: solitude coping (10 items) and solitude comfort (10 items). Solitude coping refers to one’s ability to contemplate and self-reflect when alone, whereas solitude comfort denotes whether one enjoys the time of being alone. The scale was rated on a 4-point Likert scale: 1 = *very untrue of me*, 2 = *untrue of me*, 3 = *true of me*, and 4 = *very true of me*. A high total score for the scale (or the respective subscales) indicates high capacity for solitude. This scale was verified to have satisfactory reliability and validity [32]. The Cronbach’s alpha values for the solitude capacity scale, the solitude-coping subscale, and the solitude comfort subscale were 0.86, 0.78, and 0.78, respectively.

Section 3 featured a personality trait scale. This scale was developed by Huang [34]. Referring to the Big Five model, Huang [34] divided the personality traits into five dimensions: neuroticism, extraversion, openness, agreeableness, and conscientiousness. The scale comprised 36 items and was rated on a 4-point Likert scale: 1 = *strongly disagree*, 2 = *disagree*, 3 = *agree*, and 4 = *strongly agree*. A high score indicates that the person strongly exhibits the particular personality trait, whereas a low score indicates that the person weakly exhibits the particular personality trait. The five personality traits were explained as follows: Neuroticism refers to an individual’s emotional stability, adaptability, and tendency to anxiety; individuals with a high score of neuroticism easily become sensitive, angry, and anxious, whereas those with a low score tend to be calm, relaxed, self-content, and able to see things objectively; Extraversion indicates an individuals’ behavior and its degree of manifestation in interpersonal interactions; individuals with a high extraversion score are team players, talkative, and sociable during interactions, whereas those with low scores are less likely to reveal their emotions, avoid intimate relationships with others, and tend to be submissive (which, however, does not necessarily indicate unhappiness); Openness denotes the extent to which an individual is open to experiences and her/his behavioral reactions to unfamiliar things; those with high scores are less predictable and usually think outside the box, whereas those with low scores tend to be conservative, follow traditional norms, and show a strong sense of morality; Agreeableness describes emotions expressed by an individual toward events and objects as well as during communication and interaction; it is a continuous dimension from empathy to disapproval. Individuals with a high score are warm, empathetic, cooperative, and demonstrate thoughtfulness toward others. Those with low scores tend to be judgmental, suspicious, and disagreeable; Conscientiousness is the extent to which an individual focuses on the pursuit of their goals and future achievements. Those with high scores are responsible, reliable, and adherent to moral norms in their actions; they also aim high, set challenging goals, and are highly productive. Those with low scores are more self-indulgent and spontaneous; often have unrealistic fantasies and indulge themselves in daydreams [16,34,35]. The personality trait scale exhibited satisfactory reliability and validity [35]; the Cronbach’s alpha values of the overall scale and the respective five subscales are presented in parentheses as follows: overall scale (0.85), neuroticism (0.93), extraversion (0.85), openness (0.76), agreeableness (0.75), and conscientiousness (0.86).

Section 4 was a physical and mental health scale, which measured the physical and mental statuses of the participants. Devised by Chang [36], this scale comprised 28 items and 4 subscales: somatic symptoms, anxiety and insomnia, social dysfunction, and severe depression. Somatic symptoms are physical ailments caused by excessive stress and include migraines, chronic pain, hypertension, and muscle tension. The anxiety and insomnia subscale measures symptoms such as anxiety and substandard sleep quality. Social dysfunction refers to an individual’s inability to adapt to interpersonal social interactions and work. Severe depression describes a state of hopelessness, helplessness, and even suicidal feelings. This scale was rated on a 5-point Likert scale: 1 = *not at all*, 2 = *less than usual*, 3 = *as usual*, 4 = *more than usual*, and 5 = *much more than usual*. A high score indicates an unfavorable physical and mental health status, and a low score indicates a favorable physical and mental health status. This scale was verified to demonstrate good reliability and validity [36], with the Cronbach’s alpha of four subscales presented in parentheses as follows: somatic symptoms (0.93), anxiety and insomnia (0.95), social dysfunction (0.94), and severe depression (0.95).

### 2.3. Statistical Analysis

This study employed the SPSS 22.0 for Mac (IBM Corp., Armonk, NY, USA) for data analysis. Descriptive statistics were used to reveal the participants’ demographic characteristics and descriptive results of the solitude capacity scale, personality trait scale, and physical and mental health scale. Then, Pearson’s correlation analysis was conducted to explore the correlations between the solitude capacity scale, the personality trait scale, and the physical and mental health scale. Finally, multiple regression analysis was used to verify the correlations between the solitude capacity scale, the personality trait scale, and the physical and mental health scale. In the multiple regression models, the overall score for the solitude capacity scale and the scores for the respective subscales were used as the dependent variables, and the scores for the personality trait scale and the physical and mental health scale were used as the independent variables. All demographic variables were controlled. In addition, multicollinearity diagnostics were conducted for each multiple regression model; the variance inflation factors for independent variables in all regression models were less than 10, meaning that multicollinearity could be disregarded [37].

## 3. Results

### 3.1. Participants Demography

This study recruited 562 participants (267 boys and 295 girls), whose average age was 17.51 years (SD = 1.27, range from 16 to 19 years); 2 participants were excluded due to questionnaires’ incompleteness. Table 1 presents the demographic statistics of the participants. The average body mass index (BMI) of all participants was 20.63 ± 3.66 kg/m^2^ (boys = 21.23 ± 4.13 kg/m^2^; girls = 20.18 ± 3.09 kg/m^2^), and the BMI range was from 16.30 to 41.80 kg/m^2^. Participants with no religious beliefs (57.5%) and participants who exercised on fewer than two days per week (52.7%) accounted for more than half of all the participants. Those who had a weekly allowance of less than NT$4000 (45.4%) and who lived with their families (67.8%) accounted for the largest proportion of the participants. The participants’ average total score was 56.54 ± 8.43 for the solitude capacity scale (range from 26 to 80), 28.63 ± 4.63 for the solitude-coping subscale (range from 11 to 40), and 27.92 ± 4.56 for the solitude comfort subscale (range from 10 to 40). The five personality traits in descending order of total subscale scores were as follows: conscientiousness (23.90 ± 3.72, range from 8 to 32), neuroticism (22.05 ± 6.20, range from 9 to 36), openness (19.25 ± 2.57, range from 12 to 27), extraversion (17.85 ± 3.39, range from 6 to 24), and agreeableness (17.43 ± 2.28, range from 6 to 24). The scores for the four elements of the physical and mental health scale were as follows: somatic symptoms (9.16 ± 4.85, range from 6 to 30), anxiety and insomnia (14.36 ± 7.77, range from 8 to 40), social dysfunction (10.56 ± 5.61, range from 6 to 30), and severe depression (12.09 ± 6.55, range from 8 to 40).

### 3.2. Pearson Correlation Coefficient Analysis

The Pearson’s correlation coefficients between the solitude capacity scale, the personality trait scale, and the physical and mental health scale are presented in Table 2. The total score for the solitude capacity scale was significantly correlated with four of the personality trait dimensions: neuroticism (r = −0.15, *p* < 0.01), openness (r = 0.13, *p* < 0.01), agreeableness (r = 0.13, *p* < 0.01), and conscientiousness (r = 0.16, *p* < 0.01). This score also demonstrated significant correlations with three of the physical and mental health elements: somatic symptoms (r = −0.08, *p* < 0.05), social dysfunction (r = −0.10, *p* < 0.05), and severe depression (r = −0.12, *p* < 0.01). Furthermore, in the solitude capacity scale, the solitude-coping subscale was significantly correlated with three of the personality trait dimensions: openness (r = 0.09, *p* < 0.05), agreeableness (r = 0.09, *p* < 0.05), and conscientiousness (r = 0.17, *p* < 0.01). The solitude comfort subscale exhibited significant correlations with four of the personality trait elements: neuroticism (r = −0.27, *p* < 0.01), openness (r = 0.15, *p* < 0.01), agreeableness (r = 0.16, *p* < 0.01), and conscientiousness (r = 0.12, *p* < 0.01). This subscale also demonstrated significant correlations with all physical and mental health components: somatic symptoms (r = −0.13, *p* < 0.01), anxiety and insomnia (r = −0.15, *p* < 0.01), social dysfunction (r = −0.15, *p* < 0.01), and severe depression (r = −0.16, *p* < 0.01).

### 3.3. Multiple Regression Analysis

Controlling for the demographic variables, multiple regression analysis results of the scores for the solitude capacity scale, personality traits subscales, and physical and mental health subscales were obtained and are shown in Table 3. The total score of the solitude capacity scale was significantly correlated with four of the personality traits dimensions (R^2^ = 0.11, F = 5.00, df1 = 14, df2 = 547, *p* < 0.01 ): neuroticism (B = −0.19, *p* < 0.01), extraversion (B = −0.84, *p* < 0.01), openness (B = 0.35, *p* < 0.05), and conscientiousness (B = 0.63, *p* < 0.01). As such, participants with a high solitude capacity demonstrated a high level of emotional stability, tended not to express their emotions, were less likely to build intimate relationships with others, were open to unfamiliar things, and were ambitious and goal-oriented. The solitude capacity scale demonstrated nonsignificant correlations with all physical and mental health elements.

Table 4 illustrates the results regarding the relationship of the solitude capacity subscales with the personality trait dimensions and the physical and mental health elements. The solitude-coping subscale was significantly correlated (R^2^ = 0.08, F = 3.57, df1 = 14, df2 = 547, *p* < 0.01) with extraversion (B = −0.41, *p* < 0.01) and conscientiousness (B = 0.41, *p* < 0.01). Specifically, those with an excellent ability to cope with solitude tended not to express their emotions, avoided intimate relationships with others, and were ambitious and goal-oriented. The solitude-coping subscale was significantly correlated with two of the physical and mental health elements, i.e., anxiety and insomnia (B = 0.15, *p* < 0.05) and severe depression (B = −0.14, *p* < 0.05). The results indicate that those most able to cope with solitude were prone to anxiety and insomnia but less likely to feel hopeless or helpless toward life or to feel suicidal. The solitude comfort subscale was significantly correlated (R^2^ = 0.16, F = 7.24, df1 = 14, df2 = 547, *p* < 0.01) with four of the personality trait elements, i.e., neuroticism (B = −0.18, *p* < 0.01), extraversion (B = −0.43, *p* < 0.01), openness (B = 0.25, *p* < 0.01), and conscientiousness (B = 0.23, *p* < 0.01). The results show that people exhibiting a high level of solitude comfort were emotionally stable, tended not to express their emotions, were less likely to establish intimate relationships with others, were open to unfamiliar things, and were ambitious and goal-oriented.

## 4. Discussion

This study is one of the few that have researched teenagers’ capacity for solitude, personality traits, and physical and mental health. After controlling for the demographic variables, the study results verified the association between the capacity for solitude and various personality traits. Teenagers with the traits of neuroticism and extraversion demonstrated a low capacity for solitude. By contrast, those with the traits of openness and conscientiousness demonstrated a high capacity for solitude. This result may reverse the stereotypes (stigma) of the past that teenagers who enjoy solitude have relatively negative personality traits. Moreover, the results revealed the association of the capacity for solitude with physical and mental health. Specifically, teenagers with a high solitude capacity were potentially at lower risk of physical and mental conditions. Furthermore, a high solitude-coping capacity was associated with a low level of depression but a high level of anxiety or insomnia. This implied that, although teenagers with a high solitude-coping capacity are less likely to experience severe depression, they are often burdened with anxiety. This phenomenon is supported by the personality trait results in Table 4, which indicate that teenagers with a high solitude-coping capacity are less likely to exhibit strong emotional fluctuations (low extraversion) and tend to engage in challenging activities and be goal-oriented (high conscientiousness), which may make them feel stressed and, hence, cause insomnia.

### 4.1. Correlation between Solitude Capacity and Personality Traits

Solitude capacity (including the dimensions of solitude coping and comfort) was associated with the five major personality traits. Specifically, the capacity for solitude, after controlling for demographic variables, had significantly negative correlations with neuroticism and extraversion and positive correlations with openness and conscientiousness. These results are similar to those presented by Nestler et al. (2011) and Teppers et al. (2013) [15,17]. Individuals with the trait of neuroticism frequently expressed negative emotions, experienced emotional fluctuations, and were prone to negative feelings such as discomfort and insecurity when alone. A good capacity for solitude means that one can remain emotionally stable in one’s own company, signifying a reduced probability of negative emotions occurring when one is alone and, thus, the ability to enjoy one’s time alone [38]. Accordingly, teenagers who are neurotic should learn to relax alone to relieve their intense emotions and avoid excessive stress and they may also establish a supportive social network with their family and peers.

In terms of extraversion, participants with high scores (i.e., who were active in social activities) exhibited a low solitude capacity; by contrast, those with low scores (i.e., who tended to withhold their emotions and avoid social interactions) were more easily able to spend time alone. Highly extraverted teenagers enjoy making friends and receive much support from their social networks; once they are disconnected from the outside world and are left alone, they may be unable to adapt to such situations without the company of others and may easily experience a sense of loss and lack of adaptiveness [35]. This might reflect teenagers’ adaptation to their stage of life, when they desire to be accepted by friends and seek a sense of belonging [6]. Therefore, teenagers with the trait of extraversion were encouraged to participate in courses or school clubs aimed at self-growth and leisure activities, which could enable self-improvement (including solitude capacity [34]) and a balance of their physical, mental, and social health.

Participants with high scores for openness (i.e., who were creative and open to unfamiliar things) demonstrated an extensive capacity for solitude. This indicated that teenagers who exhibit flexible thinking patterns are more likely to enjoy their own space. The association between capacity for solitude and creativity is widely supported. Isolation provides a certain type of individual freedom, which is commonly considered a necessity for improved creativity and creative activity engagement [39]. Constructive solitude (voluntary solitude) is associated with positive experiences of isolation; enjoyment of a voluntary solitude scenario and the ability to control the scenario are conducive to the growth of creativity, self-resilience, and self-understanding [33]. In addition, participants with a high score for conscientiousness (i.e., who were goal-oriented and productive) were easily able to spend time alone, meaning that ambitious teenagers enjoy their space and time alone. Such goal-oriented and productive teenagers tend to be proactive, highly autonomous and independent, resilient to frustration, able to spend time alone comfortably, and able to cope with everyday life calmly [40].

### 4.2. Correlations between Solitude Capacity and Physical and Mental Health

According to Table 4, participants with a high capacity for solitude tended to show high levels of anxiety and insomnia. This implied that, even if a teenager has a high solitude coping capacity, they do not necessarily enjoy time alone. Solitude refers to an individual being in a situation without social interaction, regardless of the place they are in [41]. Solitude can be divided into two dimensions: involuntary and constructive solitude [33]. Involuntary solitude is associated with loneliness and negative emotions, whereas constructive solitude denotes an individual’s choice to be alone and it is thus associated with a high level of autonomy [33]. Therefore, frequent engagement in involuntary solitude (i.e., isolation), even for individuals who demonstrate satisfactory solitude coping abilities, may negatively influence emotions. Furthermore, Burger (1995) [41] argued that when an individual engages in solitude to avoid crowds because of social anxiety or depression, they become socially disengaged and withdrawn, which exerts a negative influence on individuals. In addition, people who spend much time alone may be less able to adapt to new environments and be less capable of psychological and social regulation [42].

Moreover, solitude is also linked to depression; spending a long time alone often causes depression, and such negative influences are particularly prominent during engagement in involuntary solitude [33,43,44]. However, the negative correlation between solitude coping and severe depression revealed by this study means that improvement of individuals’ capacity for solitude can probably alleviate the negative influences of depression. In addition, in Taiwan, more than one-fourth of the national teenage population has experienced depressive moods (or insomnia), and the number of adolescents affected by these symptoms is increasing annually [45,46,47]. The enhancement of teenagers’ solitude capacity may be another way to improve the negative mood and/or sleep quality in these teenagers.

### 4.3. Limitation

Several limitations must be considered in the study’s interpretation of the results. First, the measurements for all scales were conducted using the participants’ self-reported information. Although these scales have all been widely used in research and exhibited satisfactory psychometric properties, they have failed to represent the solitary behaviors, personality traits, and physical and mental health of the participants which may not be measured by these tools. Second, the fact that all the participants attended the same school and that this was a period of many changes and transformations regarding personality and social relationships limited the study’s explanatory power. Third, this study was an exploratory study and focused on exploring the correlations between teenage students’ capacity for solitude and both personality traits and physical and mental health; however, there may be gender differences in the capacity for solitude. Thus, it is recommended that future research further analyze gender differences. Fourth, this study aimed to explore the correlations among teenage students’ capacity for solitude and relevant information about their personality traits and physical and mental health. Although the average age of all participants was under 18 years, a few participants were over 18 years old. Thus, this may limit the extrapolation of the findings. Fifth, this study wished to present and discuss its main results and other research results (similarities and differences). Unfortunately, no similar articles were found by using keywords in English. However, there may be other local articles that were not retrieved; thus, this limited the depth of our discussion. Sixth, although this study collected some demographic variables which might affect the participant’s solitude capacity, physical health, and mental health, it did not collect data related to the participants’ families, which might also affect the explanatory power of our models. Thus, it is recommended that future research should incorporate family variables (i.e., one-parent family). Finally, because this study adopted a cross-sectional research design, it failed to explain the causalities among the studied variables. Despite these limitations, this study revealed correlations between teenagers’ capacity for solitude and both personality traits and physical and mental health. The findings of this study may help relevant institutions and professionals to further understand the influence of teenagers’ solitude capacity on their physical and mental health.

## 5. Conclusions

Solitude is a developmental milestone that most teenagers must face and manage in their growth [15]. Solitary experiences differ across life stages. Individuals accumulate solitary experiences from their birth to their late adulthood and spend increasing time alone as they age [42]. Accordingly, people must be equipped with a high solitude capacity. According to the study results, teenagers’ capacity for solitude was associated with some of their personality traits and was not associated with negative personality traits (i.e., neuroticism). This result may reverse the stigma of the past that teenagers who enjoy solitude have relatively negative personality traits. Furthermore, the solitude coping ability was correlated positively with anxiety levels and negatively with depression. Therefore, future research should further investigate the causal relationship between individuals’ capacity for solitude and their physical and mental health.

## Figures and Tables

**Table 1:** Demography of the participants.

Demographic Variables	Total
	*N* = 562
Sex Male Female	267 (47.5%) 295 (52.5%)
Age (mean ± SD)	17.51 ± 1.27
BMI (mean ± SD)	20.68 ± 3.66
Religion (n, %)	
No	323 (57.5%)
Yes	239 (42.5%)
Exercise per week 0–1 day 2–3 days ≥4 days	296 (52.7%) 188 (33.5%) 78 (13.9%)
Money that can be spent each month	
<4000 NTD	255 (45.4%)
4000–5999 NTD	136 (24.2%)
6000–7999 NTD	54 (9.6%)
≥8000 NTD	117 (20.8%)
Have a boy/girlfriend	
No Yes	365 (64.9%) 197 (35.1%)
Living place	
Home	381 (67.8%)
School dormitory	54 (9.6%)
Off-campus rental house	127 (22.6%)

SD: Standard Deviation; BMI: Body Mass Index; NTD: New Taiwan Dollars (1000 NTD = 34 USD).

**Table 2:** Correlation coefficients between the Solitude Capacity Scale and the Personality Traits Scale as well as the Physical and Mental Health Scale.

Variable	Solitude Capacity Scale Total Score	Solitary Coping Subscale	Solitary Comfort Subscale
Personality Traits Scale			
Neuroticism	−0.15 **	−0.01	−0.27 **
Extraversion	−0.01	−0.01	−0.01
Openness	0.13 **	0.09 *	0.15 **
Agreeableness	0.13 **	0.09 *	0.16 **
Conscientiousness	0.16 **	0.17 **	0.12 **
Physical and Mental Health Scale			
Somatic symptoms	−0.08 *	−0.02	−0.13 **
Anxiety and Insomnia	−0.07	0.01	−0.15 **
Social dysfunction	−0.10 *	−0.03	−0.15 **
Severe depression	−0.12 **	−0.06	−0.16 **

* *p* < 0.05, ** *p* < 0.01.

**Table 3:** Multiple regression analysis to identify the correlation between the Solitude Capacity Scale ^†^ total score and the Personality Traits Scale, as well as the Physical and Mental Health Scale.

Independent Variable	Solitude Capacity Scale (Total Score)							
	R^2^	Adjusted R^2^	F	B	SE	Beta	95% CI	*p*
Personality Traits Scale	0.11	0.09	5.00					
Neuroticism				−0.19	0.06	−0.14	−0.31, −0.08	<0.01 **
Extraversion				−0.84	0.16	−0.34	−1.16, −0.53	<0.01 **
Openness				0.35	0.18	0.11	0.00, 0.69	0.05 *
Agreeableness				0.31	0.22	0.08	−0.13, 0.74	0.17
Conscientiousness				0.63	0.15	0.28	0.34, 0.92	<0.01 **
Physical and Mental Health Scale	0.04	0.02	1.78					
Somatic symptoms				0.02	0.14	0.01	−0.27, 0.30	0.91
Anxiety and Insomnia				0.14	0.11	0.13	−0.08, 0.36	0.22
Social dysfunction				−0.11	0.15	−0.07	−0.41, 0.20	0.49
Severe depression				−0.21	0.12	−0.16	−0.44, 0.03	0.09

^†^ Controlled for sex, age, BMI, religion, exercise per week, money that can be spent each month, having a boy/girlfriend, and living place; * *p* < 0.05; ** *p* < 0.01; B: regression coefficient; S.E.: standard error; CI: confidence interval.

**Table 4:** Multiple regression analysis identifying a significant correlation between the Solitude Capacity Scale ^†^ and two dimensions of the Personality Traits Scale and the Physical and Mental Health Scale.

Independent Variable	Solitary Coping Subscale															
	R^2^	A R^2^	F	B	SE	Beta	95% CI	*p*	R^2^	AR^2^	F	B	SE	Beta	95% CI	*p*
**Personality Traits Scale**	0.08	0.06	3.56						0.16	0.13	7.19					
**Neuroticism**				−0.01	0.03	−0.02	−0.08, 0.06	0.74				−0.18	0.03	−0.25	−0.25, −0.12	<0.01 **
**Extraversion**				−0.41	0.09	−0.30	−0.59, −0.23	<0.01 **				−0.43	0.09	−0.32	−0.60, −0.27	<0.01 **
**Openness**				0.09	0.10	0.05	−0.10, 0.29	0.33				0.25	0.09	0.14	0.07, 0.43	<0.01 **
**Agreeableness**				0.10	0.12	0.05	−0.14, 0.35	0.40				0.20	0.12	0.10	−0.03, 0.43	0.08
**Conscientiousness**				0.41	0.08	0.33	0.24, 0.57	<0.01 **				0.23	0.08	0.19	0.08, 0.38	<0.01 **
**Physical and Mental Health Scale**	0.03	0.01	1.44						0.06	0.04	2.60					
**Somatic symptoms**				0.01	0.08	0.01	−0.15, 0.16	0.95				0.01	0.08	0.01	−0.14, 0.16	0.89
**Anxiety and Insomnia**				0.15	0.06	0.24	0.02, 0.27	0.02 *				−0.01	0.06	−0.01	−0.12, 0.11	0.91
**Social dysfunction**				−0.06	0.09	−0.08	−0.23, 0.10	0.45				−0.04	0.08	−0.05	−0.20, 0.12	0.61
**Severe depression**				−0.14	0.07	−0.20	−0.27, −0.01	0.04 *				−0.07	0.07	−0.10	−0.20, 0.06	0.31

^†^ Controlled for sex, age, BMI, religion, exercise per week, money that can be spent each month, having a boy/girlfriend, and living place; * *p* < 0.05; ** *p* < 0.01 B: regression coefficient; S.E.: standard error; CI: confidence interval. AR: Adjusted R^2.^

## References

[1] Larson R.W., Raffaelli M., Richards M.H., Ham M., Jewell L. (1990). Ecology of depression in late childhood and early adolescence: A profile of daily states and activities. J. Abnorm. Psychol..

[2] Larson R., Csikszentmihalyi M., Graef R. (1982). Time alone in daily experience: Loneliness or renewal. Loneliness: A Sourcebook of Current Theory, Research and Therapy.

[3] Bruno S., Lutwak N., Agin M.A. (2009). Conceptualizations of guilt and the corresponding relationships to emotional ambivalence, self-disclosure, loneliness and alienation. Personal. Individ. Differ..

[4] Goosby B.J., Bellatorre A., Walsemann K.M., Cheadle J.E. (2013). Adolescent loneliness and health in early adulthood. Sociol. Inq..

[5] Cornwell E.Y., Waite L.J. (2009). Social disconnectedness, perceived isolation, and health among older adults. J. Health Soc. Behav..

[6] Brennan T. (1982). Loneliness at Adolescence. Teoksessa LA Peplau & D. Perlman (toim.). Loneliness: A Sourcebook of Current Theory, Research and Therapy.

[7] Terrell-Deutsch B. (1999). 2| The Conceptualization and Measurement. Loneliness in Childhood and Adolescence.

[8] Hawkley L.C., Cacioppo J.T. (2010). Loneliness matters: A theoretical and empirical review of consequences and mechanisms. Ann. Behav. Med..

[9] Goossens L., Marcoen A. (1999). Adolescent Loneliness, Self-Reflection, and Identity: From Individual Differences to Developmental Processes.

[10] Corsano P., Majorano M., Champretavy L. (2006). Psychological well-being in adolescence: The contribution of interpersonal relations and experience of being alone. Adolescence.

[11] Buchholz E.S., Catton R. (1999). Adolescents’ perceptions of aloneness and loneliness. Adolescence.

[12] Goossens L., Lasgaard M., Luyckx K., Vanhalst J., Mathias S., Masy E. (2009). Loneliness and solitude in adolescence: A confirmatory factor analysis of alternative models. Personal. Individ. Differ..

[13] Wang J.M. (2016). Preference-for-solitude and depressive symptoms in Chinese adolescents. Personal. Individ. Differ..

[14] Asendorpf J.B., Van Aken M.A. (2003). Personality–relationship transaction in adolescence: Core versus surface personality characteristics. J. Personal..

[15] Teppers E., Klimstra T.A., Damme C.V., Luyckx K., Vanhalst J., Goossens L. (2013). Personality traits, loneliness, and attitudes toward aloneness in adolescence. J. Soc. Pers. Relatsh..

[16] McCrae R.R., Costa P.T. (2003). Personality in Adulthood: A Five-Factor Theory Perspective.

[17] Nestler S., Back M.D., Egloff B. (2011). Psychometric properties of the two scales for assessing individual differences in preference for solitude. Diagnostica.

[18] Cacioppo J.T., Hawkley L.C., Ernst J.M., Burleson M., Berntson G.G., Nouriani B., Spiegel D. (2006). Loneliness within a nomological net: An evolutionary perspective. J. Res. Personal..

[19] Russell D., Peplau L.A., Cutrona C.E. (1980). The revised UCLA Loneliness Scale: Concurrent and discriminant validity evidence. J. Personal. Soc. Psychol..

[20] Long C.R., Seburn M., Averill J.R., More T.A. (2003). Solitude experiences: Varieties, settings, and individual differences. Personal. Soc. Psychol. Bull..

[21] Tsai J.-T. (2014). Solitude Preference and Ability to Be Alone in Adolescent: The Moderating Effect of Mindfulness.

[22] Savikko N., Routasalo P., Tilvis R.S., Strandberg T.E., Pitkälä K.H. (2005). Predictors and subjective causes of loneliness in an aged population. Arch. Gerontol. Geriatr..

[23] Heinrich L.M., Gullone E. (2006). The clinical significance of loneliness: A literature review. Clin. Psychol. Rev..

[24] Stickley A., Koyanagi A., Koposov R., Blatný M., Hrdlička M., Schwab-Stone M., Ruchkin V. (2016). Loneliness and its association with psychological and somatic health problems among Czech, Russian and US adolescents. Bmc Psychiatry.

[25] Eccles A.M., Qualer P., Madsen K.R., Holstein B.E. (2020). Loneliness in the lives of Danish adolescents: Associations with health and sleep. Scand. J. Public Health.

[26] Sharma B., Lee T.H., Nam E.W. (2017). Loneliness, insomnia and suicidal behavior among school-going adolescents in Western Pacific Island countries: Role of violence and injury. Int. J. Environ. Res. Public Health.

[27] Sauter S.R., Kim L.P., Jacobsen K.H. (2020). Loneliness and friendlessness among adolescents in 25 countries in Latin America and the Caribbean. Child Adolesc. Ment. Health.

[28] Zhou J., Li X., Tian L., Huebner E.S. (2020). Longitudinal association between low self-esteem and depression in early adolescents: The role of rejection sensitivity and loneliness. Psychol. Psychother. Theory Res. Pract..

[29] Okwaraji F.E., Obiechina K.I., Onyebueke G.C., Udegbunam O.N., Nnadum G.S. (2018). Loneliness, life satisfaction and psychological distress among out-of-school adolescents in a Nigerian urban City. Psychol. Health Med..

[30] De Oliveira Costa R.P., Peixoto A.L.R.P., Peixoto C.C.A.L., Falcão D.N., da Silva Farias J.T., Viana L.F.P., de Alencar Pereira M.A., Sandes M.L.B., Lopes T.B., Mousinho K.C. (2020). Profile of non-suicidal self-injury in adolescents: Interface with impulsiveness and loneliness. J. Pediatr..

[31] Herz M., Lalander P. (2017). Being alone or becoming lonely? The complexity of portraying ‘unaccompanied children’as being alone in Sweden. J. Youth Stud..

[32] Wu L.-C., Chen S.-F. (2006). Relationships among the Ability to be Alone, Subjective Life Stress and Mental Health in Junior gh School Students. Bull. Ofeducational Psychol..

[33] Larson R., Lee M. (1996). The capacity to be alone as a stress buffer. J. Soc. Psychol..

[34] Huang Y.-T. (2006). A Study of Teacher’s Personality Traits Effect on Organizational Citizenship Behavior—Taking the Public Junior High Schools in Nantou County for Example.

[35] Hsieh Y.-T. (2014). A Study on Singles’ Personality Traits and Capacity to be Alone toward Physical and Mental Health in Taipei Area.

[36] Chang C. (1987). Study on University Entrance Examination Stress Syndrome. Taiwan J. Public Health.

[37] Marquandt D. (1980). You should standardize the predictor variables in your regression models. Discussion of: A critique of some ridge regression methods. J. Am. Stat. Assoc..

[38] Creswell K.G., Chung T., Wright A.G., Clark D.B., Black J.J., Martin C.S. (2015). Personality, negative affect coping, and drinking alone: A structural equation modeling approach to examine correlates of adolescent solitary drinking. Addiction.

[39] Amabile T.M. (2018). Creativity in Context: Update to the Social Psychology of Creativity.

[40] Yang K.-S., Lu L. (2005). Social- and Individual-oriented Self-actualizers: Conceptual Analysis and Empirical Assessment of their Psychological Characteristics. Indig. Psychol. Res. Chin. Soc..

[41] Burger J.M. (1995). Individual differences in preference for solitude. J. Res. Personal..

[42] Larson R.W. (1990). The solitary side of life: An examination of the time people spend alone from childhood to old age. Dev. Rev..

[43] Storr A. (2005). Solitude: A Return to the Self.

[44] Lasgaard M., Goossens L., Elklit A. (2011). Loneliness, depressive symptomatology, and suicide ideation in adolescence: Cross-sectional and longitudinal analyses. J. Abnorm. Child Psychol..

[45] Yang S.-Y., Fu S.-H., Chen K.-L., Hsieh P.-L., Lin P.-H. (2019). Relationships between depression, health-related behaviors, and internet addiction in female junior college students. PLoS ONE.

[46] Wang P.-Y., Chen K.-L., Yang S.-Y., Lin P.-H. (2019). Relationship of sleep quality, smartphone dependence, and health-related behaviors in female junior college students. PLoS ONE.

[47] Lin P.-H., Lee Y.-C., Chen K.-L., Hsieh P.-L., Yang S.-Y., Lin Y.-L. (2019). The Relationship Between Sleep Quality and Internet Addiction Among Female College Students. Front. Neurosci..

